# Status of chemistry lab safety in Nepal

**DOI:** 10.1371/journal.pone.0179104

**Published:** 2017-06-23

**Authors:** Krishna Prasad Kandel, Bhanu Bhakta Neupane, Basant Giri

**Affiliations:** 1 Department of Chemistry, Birendra Multiple Campus, Tribhuvan University, Chitwan, Nepal; 2 Center for Analytical Sciences, Kathmandu Institute of Applied Sciences, Kathmandu, Nepal; 3 Department of Chemistry, Amrit Campus, Tribhuvan University, Kathmandu, Nepal; Holbæk Hospital, DENMARK

## Abstract

Chemistry labs can become a dangerous environment for students as the lab exercises involve hazardous chemicals, glassware, and equipment. Approximately one hundred thousand students take chemistry laboratory classes annually in Nepal. We conducted a survey on chemical lab safety issues across Nepal. In this paper, we assess the safety policy and equipment, protocols and procedures followed, and waste disposal in chemistry teaching labs. Significant population of the respondents believed that there is no monitoring of the lab safety in their lab (p<0.001). Even though many labs do not allow food and beverages inside lab and have first aid kits, they lack some basic safety equipment. There is no institutional mechanism to dispose lab waste and chemical waste is disposed haphazardly. Majority of the respondents believed that the safety training should be a part of educational training (p = 0.001) and they would benefit from short course and/or workshop on lab safety (p<0.001).

## Introduction

The chemistry teaching labs, where students perform experiments using chemicals to test the theoretical concept they learn in classes, are crucial in learning chemistry. Further, laboratory exercises help to increase students’ interest towards chemistry. However, hazardous chemicals, glassware, and equipment in the labs may pose dangerous environment to students and instructors in absence of proper safety measures. A proper chemical hygiene plan (CHP) [1] is always important to minimize injuries, which can be life-threatening in some cases. It is well said that the accidents in chemistry lab do not happen; rather they are caused and are avoidable in many circumstances. Most of the dangers in lab can be avoided with careful planning [1].

A proper CHP includes several measures such as proper storage of chemicals, safe and proper handling of chemicals and glassware, proper design of experiments, safer physical facilities and equipment, and appropriate chemical waste management system [1]. Furthermore, providing proper safety training to teachers and students can minimize risk in chemistry lab. In addition, appropriate regulations and regular monitoring in place from government agencies are also crucial. While the lab safety is top most priority in developed countries, it is often neglected in developing countries. The developing countries lack either legal framework for laws and regulations for chemical safety or lack effective enforcement or both [2]. Furthermore, it is believed that teaching chemistry labs in these countries have relatively large number of students in one session thus increasing the risk. A number of previous studies carried out in schools and universities in developing countries (the Philippines [3], African countries [4], Iran [5]) have indicated a poor safety and security conditions.

Chemistry teaching started in 1919 AD for the first time in Nepal at Trichandra Multiple College, Tribhuvan University [6]. Currently, chemistry is being taught to about forty-six thousand students annually in about six hundred high schools (11^th^ and 12^th^ grade) across Nepal. All these schools follow same examination board (previously higher education council) under ministry of education, government of Nepal. In addition, our rough estimate shows that approximately sixty-five thousand students take chemistry courses every year in undergraduate and graduate programs under eight universities and four academies [7]. However, there are no reports and publications that evaluated the safety in chemistry labs in Nepal.

In this paper, we aim to evaluate the overall safety status of chemistry teaching labs in Nepal. We conducted a survey among chemistry teachers hoping to help high schools, universities, and government agencies to put and enforce appropriate safety practices and regulation. Our results show that the labs lack some of the basic safety equipment and regulation from government agencies. The respondents agreed to the fact that safety training must be provided to them. At the end of this paper, we provide some recommendations that are helpful for the safe handling of chemistry labs and for the safety of students and teachers in these labs.

## Methodology

We conducted a survey among chemistry teachers in Nepal. The questionnaire was designed to gather information on general safety equipment and procedures followed, waste handling and disposal, and teachers’ training on safety issues in Nepal. At first, the survey questionnaires were distributed among chemistry teachers in Chitwan district. Based on the response we obtained, the questions were revised for clarity and then later we reached out to the chemistry teachers in other parts of the country (see S1 File for the questionnaire). Anonymity of the teacher and school/college was maintained during the survey. The safety survey entitled “chemistry laboratory safety in Nepal” consisted of 70 open- and closed-ended questions [8, 9]. The questions were divided into following sections: general laboratory safety procedures and safety equipment, chemical storage and procurement system, waste disposable, teacher safety training, and safety policy. The questions were made available via Google form and the link of the form was shared via email, Facebook messages, and social networking platform of Nepali chemists. The survey questions were also printed and distributed to the teachers who did not have easy access to the Internet. Written explanation regarding the purpose of the study along with the survey questions was provided to the potential respondents to help them with decision making on whether or not to participate. Among about 782 chemistry teaching labs across Nepal, we reached out to ~250 potential respondents and received 138 responses. Responses were only included from individuals who said they were teaching chemistry labs. The online survey responses were obtained in excel sheet whereas responses obtained off-line were also entered into the excel sheet manually.

## Results and discussion

### Chemistry labs in Nepal

The respondents of the survey represented 19 districts out of 75 districts in Nepal. The respondents were from Banke, Bara, Bhaktapur, Chitwan, Dang, Dhankuta, Jhapa, Kailali, Kaski, Kathmandu, Kavre, Lalitpur, Makwanpur, Morang, Nawalparasi, Palpa, Rupandehi, Sunsari, and Surkhet districts of Nepal. These districts are major educational hubs in Nepal and represent more than 86% of science teaching higher education institutions [7].

**Fig 1:**
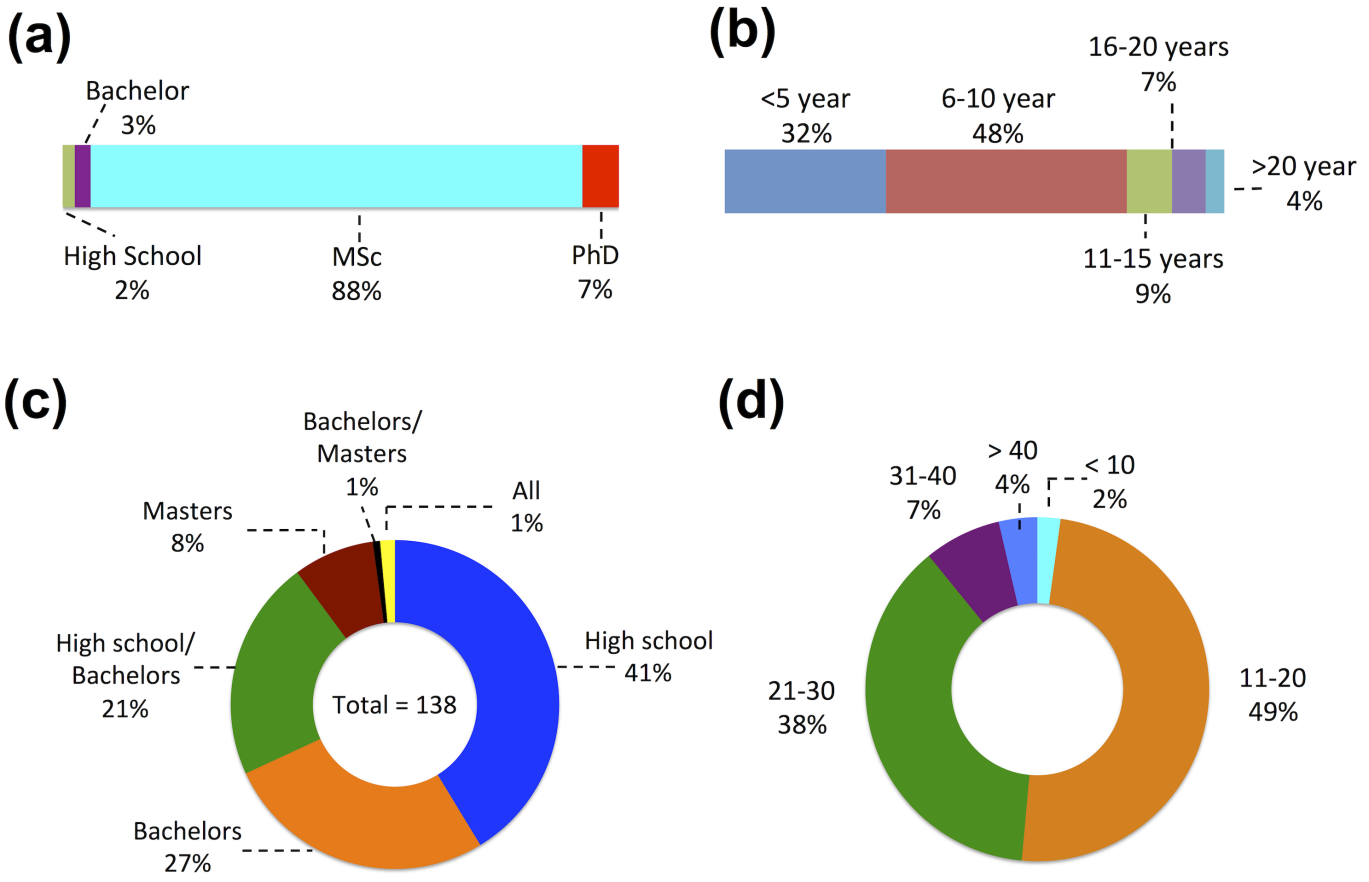
(a) Highest education degree of respondents, (b) Respondents’ teaching experience duration, (c) Level of courses offered in the chemistry labs represented in this survey, and (d) Number of students in each lab session.

The academic degree of the respondents varied widely. It ranged from high school (2%) to PhD (7%). However, majority of the respondents, as shown in Fig 1a, had MSc degree (88%). Education system in Nepal requires at least a master’s degree to be eligible as a faculty both in high school (11^th^& 12^th^ grade) and in university. The respondents (5%) in this survey with only high school or bachelor’s degree may have been working as lab assistants in respective chemistry labs. Duration of teaching experience of the respondents also ranged quite a bit. It ranged from less than six years (32%) to more than 20 years (4%). Majority of the respondents (68%) had more than 6 years (Fig 1b) of experience in teaching chemistry lab sessions.

Out of 138 respondents, 41% said that the chemistry lab in their institution is used only for higher secondary level (Fig 1c). Higher secondary level in Nepal includes11^th^ and 12^th^grades affiliated to higher secondary education board (now National Examination Board), government of Nepal. General certification of education advanced level (A-level) affiliated to secondary school leaving qualification in the United Kingdom is also included in this category. Similarly, 27% said their lab is used for bachelor level courses, whereas 21% said the lab is used for both high school and bachelor level. This shows that the data collected from our survey represents major sectors of chemistry teaching labs in Nepal. The number of students worked in each lab session ranged from less than 10 (2%) to more than 40 (4%). Majority of labs had either 11–20 students (49%) or 21–30 (38%) students in each session (Fig 1d). The majority (51%) of respondents said their lab had two instructors including lab assistants in each lab session, whereas 30% said three teaching staffs, 12% said only one staff and remaining 7% respondents said they had four staffs during the lab session. Furthermore, the higher proportion of the chemistry labs (44%), as reported in our survey, was 21–40 m^2^ sizes followed by 20% being >80 m^2^, 19% 41–60 m^2^, 9% 61–80 m^2^ and 8% <20 m^2^.

### Laboratory safety equipment and practice

**Fig 2:**
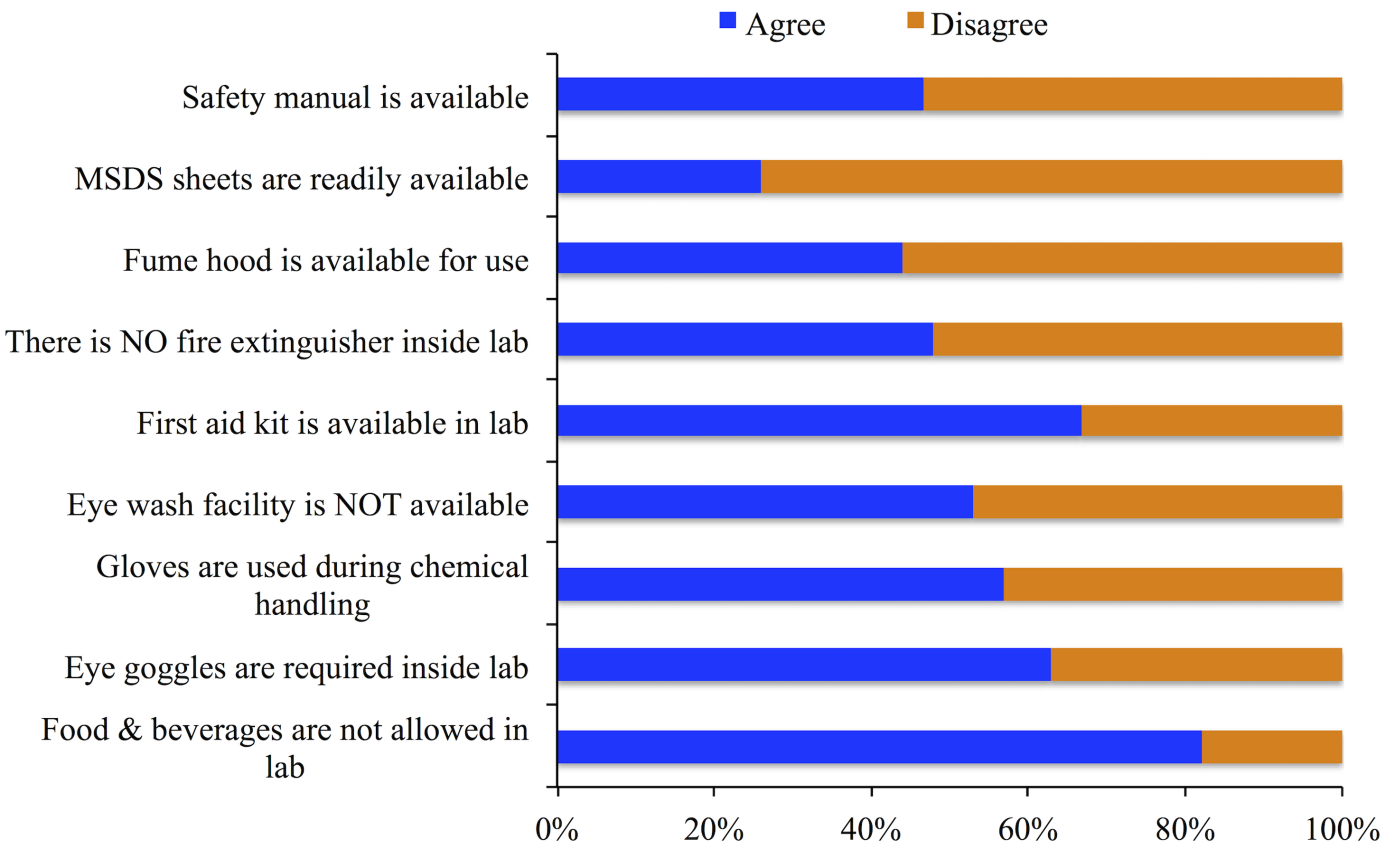
Responses to various lab safety equipment and practices questions.

Availability and use of personal protective equipment such as safety goggles, lab coat, fire extinguisher, gloves, eye fountain etc. and other equipment such as fume hood play crucial role to protect the lab users from any chemical hazards [10]. In addition, procedures and protocols followed inside lab are also important in maintaining lab as a safe place. Majority of the respondents mentioned that their institution (82%, p<0.001) does not allow food and beverages inside the lab. Furthermore, 63% of the respondents said students use safety goggles and 57% said that gloves are used while handling chemicals. However, only 46% chemistry teaching labs have one or the other form of eye wash station facilities. We speculate, from our visit to some of the labs and also based on our own experience, these eye wash stations may be just regular house bathroom basin and taps that may not serve as the effective eye wash fountain. In addition, relatively large numbers (67%) of lab keep first aid kit in the lab. Number of labs possessing fire extinguishers, fume hood, the material safety data sheet (MSDS), and safety manual are 52%, 44%, 26%, and 47%, respectively (*see* Fig 2). Most importantly, the condition of fire extinguishers is rarely monitored by external agencies and school/university itself.

### Storage of chemicals

**Fig 3:**
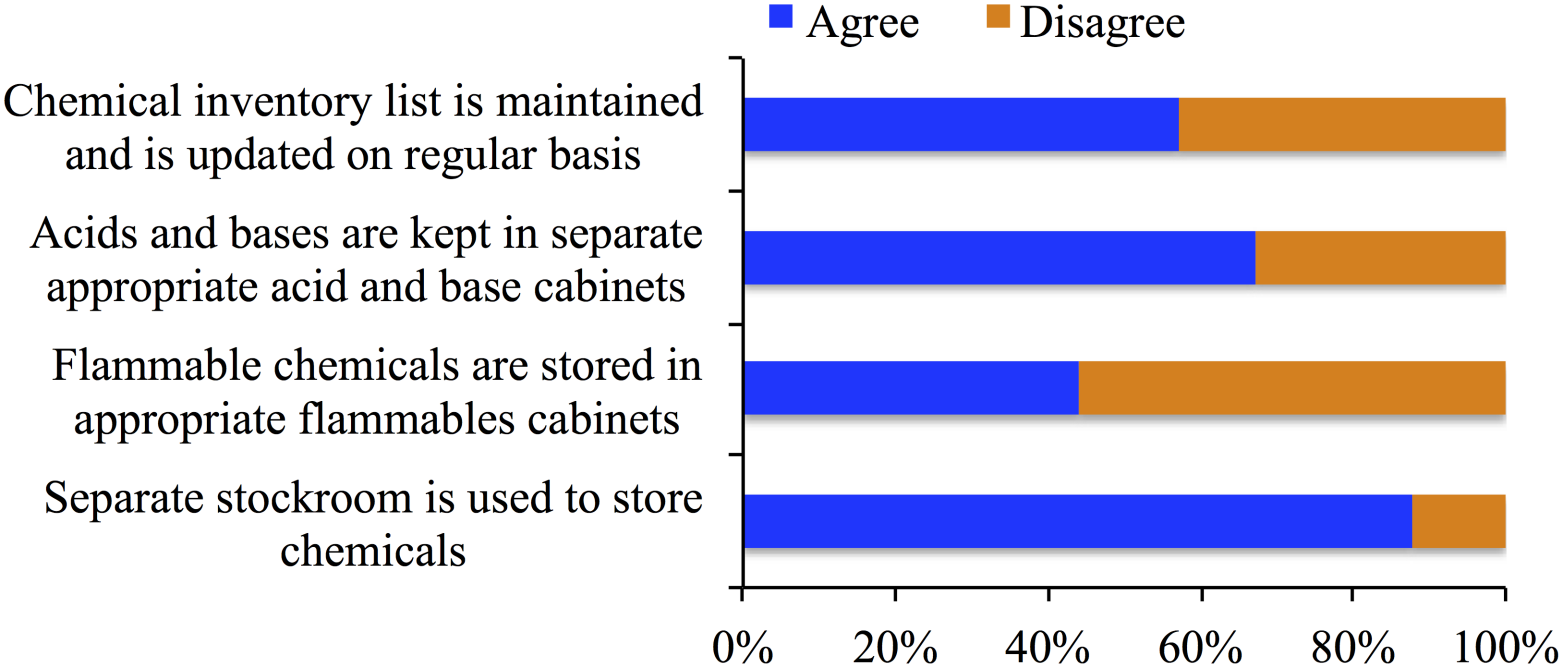
Responses to questions related to chemical storage practices.

It is essential to have separate stock room for storage of chemicals and should not be accessible to the students. Separate storage according to hazard category and compatibility is also necessary [2]. We asked the respondents a series of questions to know how the chemicals are stored in their labs. Our results show that most of the labs (88%) have separate stockroom for chemical storage (p<0.001). Only 57% respondents stated that a chemical inventory list is maintained in their labs. 44% respondents agreed on the statement that said separate cabinet is used for flammable chemicals and 67% agreed on the statement that said acid and base are arranged in separate compartments (*see* Fig 3).

### Emergency and accidents

**Fig 4:**
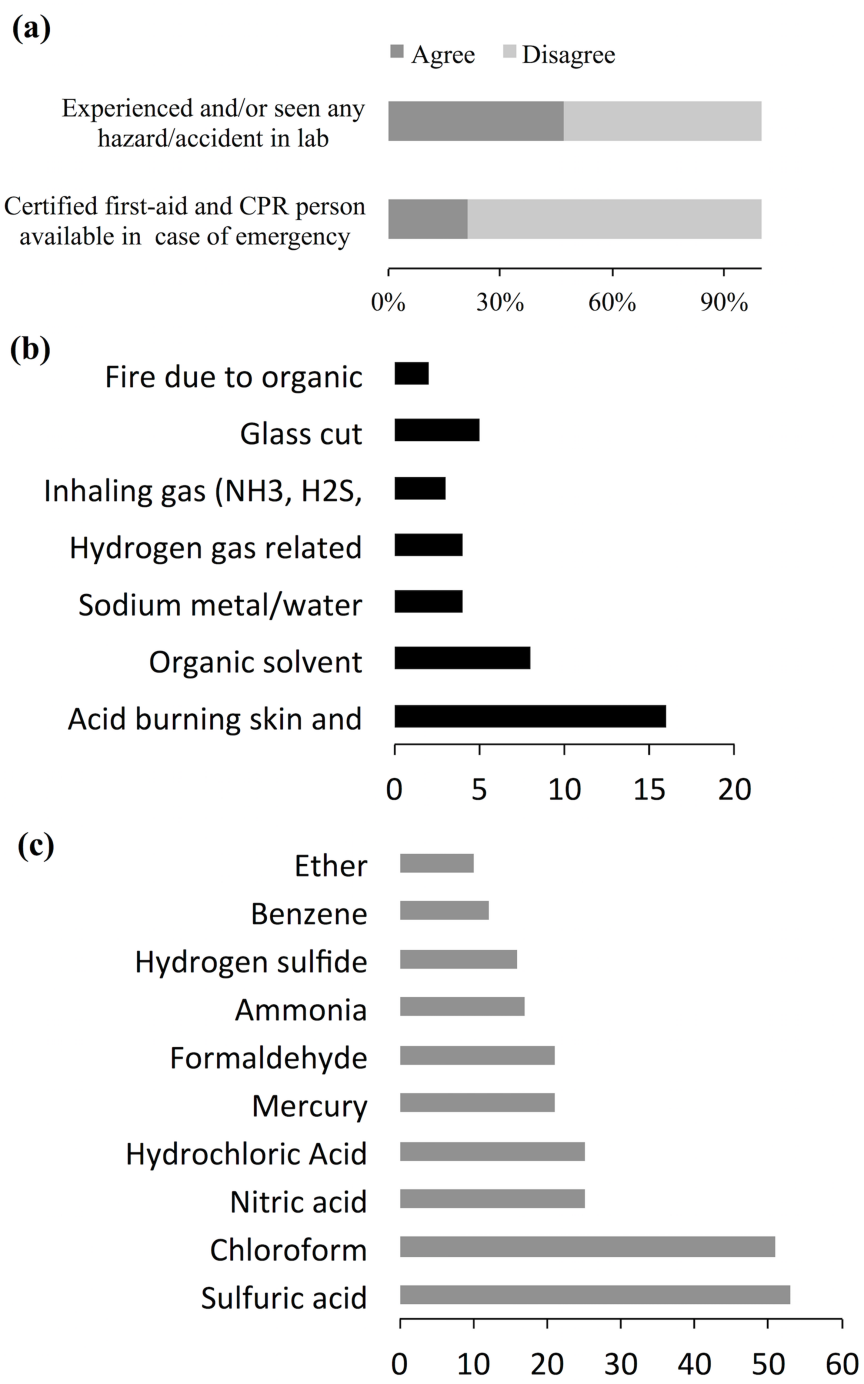
Responses on emergency handling questions. (a) Percentage of respondents that agreed and disagreed on the given statement, (b) Number of times the given accident reported, and (c) The most frequently reported hazardous chemicals per respondents’ knowledge.

Next set of questions was related to accidents, hazardous incidents, and emergency handling procedures in the chemistry teaching labs. It appears that 47% respondents had experienced or seen accidents and/or hazardous situation in the lab (Fig 4a). Respondents who answered positive in this question were asked to give examples of the hazards they came across. Out of 52 responses for examples of hazardous/accidents, the most frequent hazards [11] among reported cases resulted from improper use of acid leading to injury, mostly damaging skin and cloth (Fig 4b). The acid reported in this case was the concentrated sulfuric acid. Similarly, 8 injuries reported in the survey occurred when working with organic solvents such as benzene. Organic solvent without proper safety gears such as gloves, masks and fume hood may corrode skin, catch fire and the user may suffocate because of solvent vapor. Similarly, 4% injuries occurred when sodium metal got in contact with water leading to explosion and fire. Some of the injuries resulted while preparing and testing hydrogen gas (4), glass cut (5), and only 3 hazards were reported to be by toxic gas (e.g., NH_3_, H_2_S, and SO_2_) inhalation (Fig 4b). Among the injury and accidents reported in the survey, students were taken to the hospital only in couple of instances. One of the respondents wrote-*“During my B*. *Sc*. *chemistry lab at Butwal Multiple Campus*, *my friend got into an accident involving concentrated acid that burned her cloth and penetrated to the skin*. *We did not have first aid materials and therefore she was immediately taken to a nearby hospital*. *Now I realize that we used to use acid without having any precaution*.*”* In a situation like this, eyewash fountain, safety shower, and first aid kit in addition to a trained health worker nearby should have played an important role to minimize the injury but as described above not all labs had these facilities. The results showed, only 20% labs (Fig 4a) had somebody familiar with first aid procedure and cardiopulmonary resuscitation (CPR).

We also asked respondents to name the five most hazardous chemicals in the lab. Out of 60 chemicals reported as hazardous, the most frequently reported ten hazardous chemicals (Fig 4c) were concentrated sulfuric acid (53), chloroform (51), nitric acid (25), hydrochloric acid (25), mercury (21), formaldehyde (21), ammonia (17), hydrogen sulfide (16), benzene (12), and ether (10). Most of the respondents reported acids in their top ten hazardous chemicals list and very few report bases. Even though bases are frequently used in teaching labs and are hazardous, underreporting bases as hazardous chemical may be because of lack of understanding on the hazardous nature of chemicals. It reflects the need of training to chemistry teachers on the risk of each chemical possess.

### Chemical waste management

**Fig 5:**
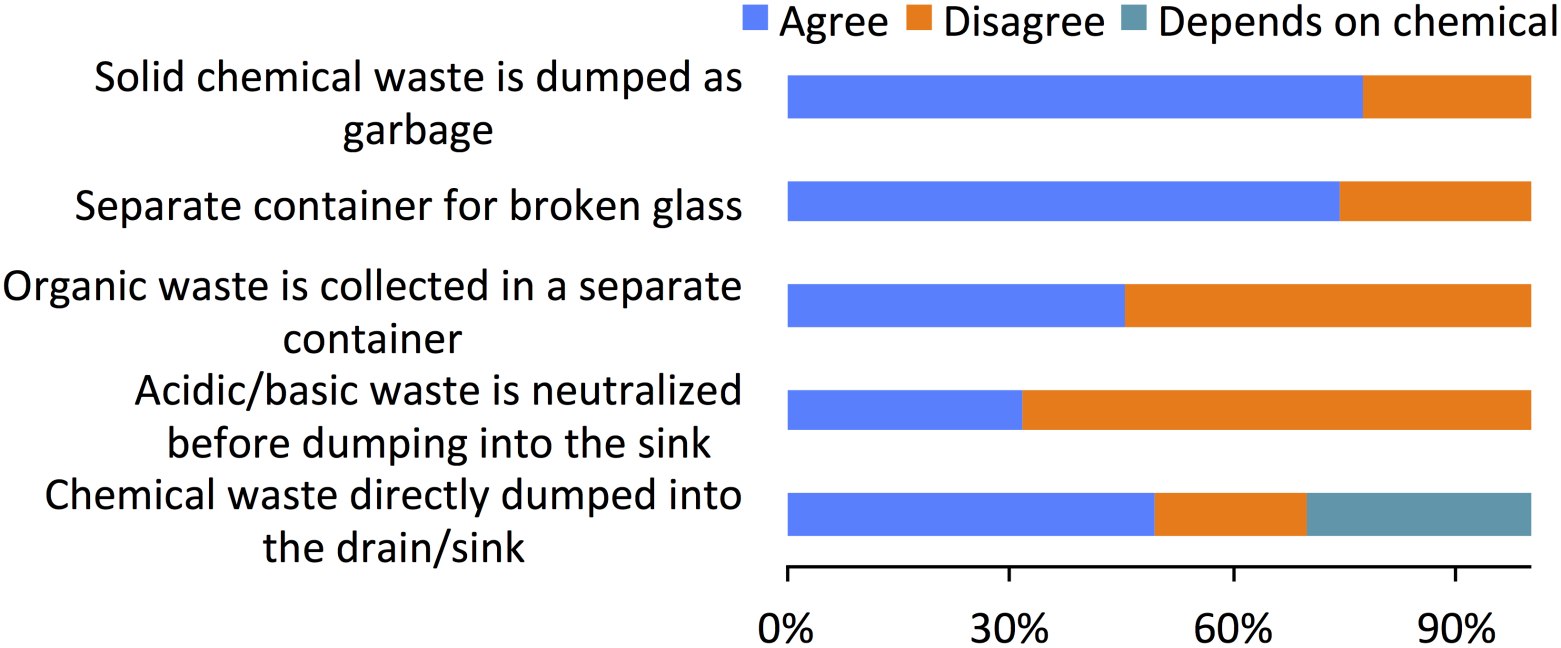
Responses to waste management questions.

Hazardous chemical waste, as designated by the United States Environmental Protection Agency (EPA) [11], is a waste that presents danger to human health and/or the environment. According to EPA regulations, there are four characteristics that define a waste as hazardous: ignitability, corrosivity, reactivity, and toxicity. In addition, there are lists of hundreds of other chemicals that EPA has determined to be hazardous waste. Our result suggests that 79% labs dispose the solid chemical waste directly into regular household waste disposal system. 49% lab users dumped liquid chemical waste directly into the drain/sink and 31% dumped according to the nature of chemical. In general practice, acid and base are neutralized before draining into the drainage system worldwide. In our research (Fig 5) we found that 68% chemistry teaching labs drain the acid and/or base waste without neutralization. Interestingly, 58% labs did not separate organic waste from other non-organic waste. However, 76% stated that they used separate container for collecting broken glass. The broken glasses are also finally dumped into regular garbage, as there are no mechanisms for disposing them separately. Solid waste management in Nepal has been a great problem since past couple of decades. Solid waste in the largest and capitol city of Nepal, Kathmandu, contains 1% hazardous waste [12]. It should be noted that every kind of waste including hazardous hospital waste is handled as regular garbage in Nepal except by few bigger hospitals that incinerate the hospital waste. There are no specialized agencies or companies for handling hazardous waste in Nepal.

### Lab safety training and policy

**Fig 6:**
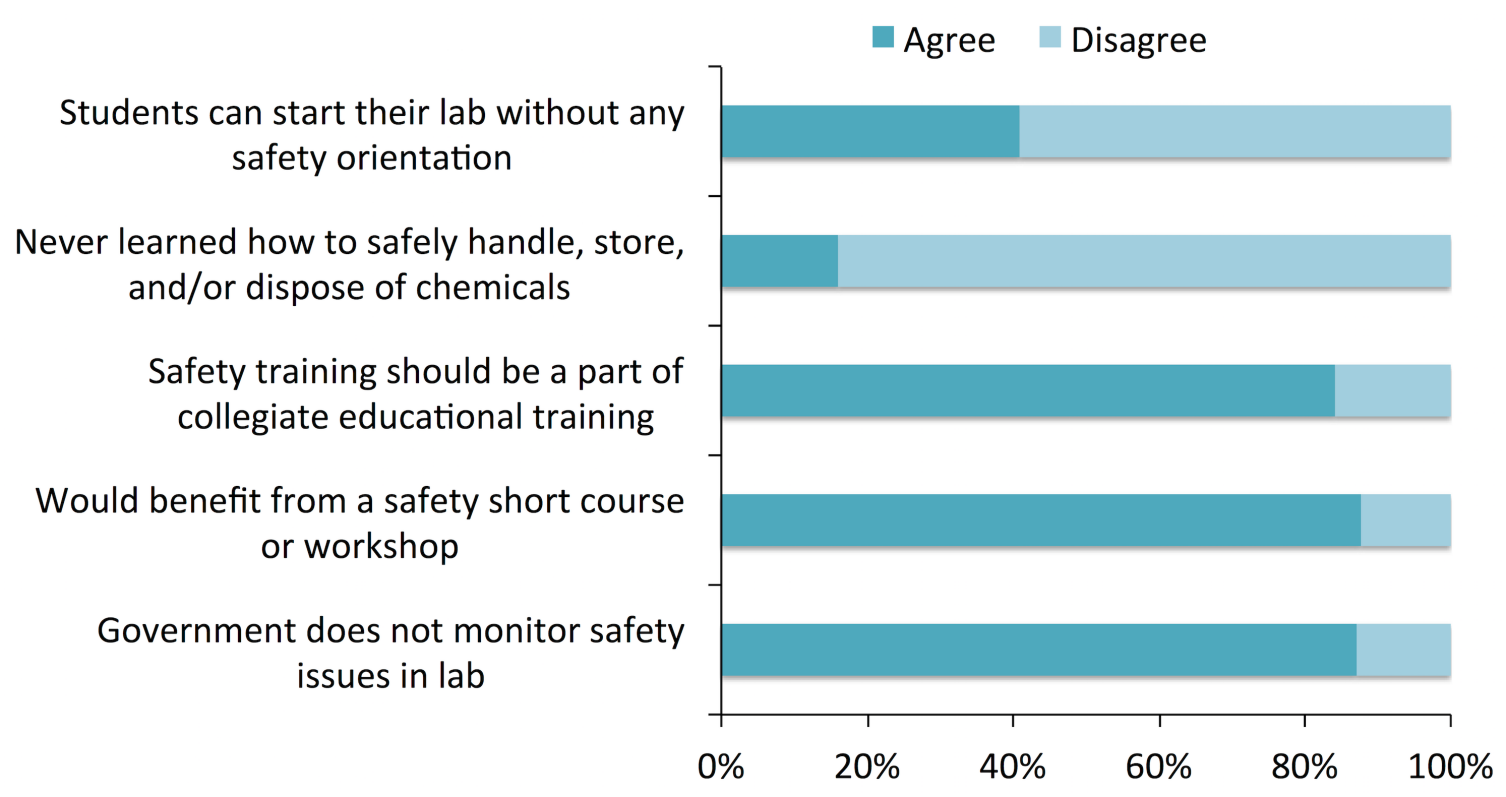
Responses to safety training and policy section.

Laboratory instructors or teaching assistants should be instructed on safe laboratory techniques and work practices. The training must start at a classroom. As suggested by Alaimo et al. [13] the first lab period of each course should be dedicated to lab safety orientation. Introduction of an independent lab safety course as a mandatory course for the student as a part of undergraduate degree should be enforced [14]. It is evident from this survey that more than 41% respondents agreed with the fact that their students can start even the first lab without any safety orientation (Fig 6), while 59% said they provide safety orientation. The extent of topics covered by such orientation is not clear. Duration of this safety orientation may range from 10–20 min briefing to an hour presentation by a faculty as is happening at the central department of chemistry, Tribhuvan University recently. However, 88% respondents stated they had some sort of safety training needed to safely handle, store, and dispose chemicals used in teaching labs. We went through the chemistry syllabus of higher secondary level (the 11^th^ and 12^th^ grade) and undergraduate and graduate levels of Tribhuvan University, Nepal. None of the courses contain safety topics and lab safety sessions. However, Tribhuvan University recently introduced a fourth year undergraduate theoretical elective course on applied chemistry in which 25 hours of instruction have been allocated for topics related to safety considerations in chemical process industries [15]. Because the safety training and orientation is not mandatory, it is the individual teachers’ will and interest to run safety orientation on the first day of lab. These facts support 84% respondent’s response for the necessity of collegiate educational safety training as a part of curriculum. 88% respondents would be interested to participate in a short course or workshop that focuses on safe handling, storage, and disposing chemicals. Most of the respondents (87%) believed there was no government agency to monitor the safety issues in teaching chemistry labs. It is interesting to note, however, that the Nepal Bureau of Standards and Metrology, Government of Nepal had prepared a safety code for chemistry laboratories in 1989 [16]. This code as a safety guideline is supposed to be implemented in both teaching and non-teaching chemistry laboratories in the country. Unfortunately, chemists we surveyed were not aware of the guidelines.

## Conclusions and recommendations

A safety program entails proper attention and contribution from various sectors such as government agencies (through policy making), and individuals, for example commitment of teachers, students, and the department and school/university officials to ensure safety in teaching labs. In this paper, we presented our results based on a questionnaire survey of chemistry teaching lab safety status among the chemistry teachers in Nepal. We showed that the chemistry labs in Nepal, in general, had issues ranging from the lack one or the other safety equipment, to disposal of chemical waste as regular waste. Despite the presence of a chemistry lab safety code prepared by Nepal government, most chemistry instructors were unaware of the code indicating a lack of monitoring in the government’s part and ignorance in the university education system. The school and university systems neither had separate lab safety policy nor their own regulating mechanism.

Whereas very little attention has been paid to this sector by the concerned authorities, an increasing realization of the importance of safety in labs and an increasing need for lab safety trainings prior to starting lab works among is observed among the students and teachers.

We recommend that the Government of Nepal must update its 20 years old code on chemistry lab safety because not only the numbers of teaching and non-teaching chemistry labs and people involved with these labs have increased drastically, but also the classes of chemicals and available equipment for maintenance have updated and added a lot since 1989 AD. Further, strict enforcing those safety codes is a most. We suggest the university grants commission of Nepal monitor the safety issues and schools/university create independent safety code enforcement body. University and schools must have their own working protocols for safety related issues and handling emergency situations. We also recommend every lab course must start with a safety session and orientation. We find it compulsory to include one mandatory safety course (at least one credit hour) as a part of undergraduate degree. Last, but not the least, lab safety training and workshops should be organized to train teachers and lab assistants on regular basis to familiarize them with latest updates in the realm of chemistry safety. Finally, as our work is based on survey, we cannot ignore the inherent error associated with survey-based research. The error may have also come from the variation in the academic qualification of the respondents.

## Supporting information

S1 File Sample questionaaire. (PDF)

S2 File Data details. (PDF)
