# Supplementary material for: Status of chemistry lab safety in Nepal
Source: PLoS One. 2017 Jun 23;12(6):e0179104. doi: 10.1371/journal.pone.0179104 (PMC5482429; doi:10.1371/journal.pone.0179104)
Supplement: S1 File — (PDF) [file pone.0179104.s001.pdf]

Status of chemistry lab safety in Nepal

Krishna Prasad Kandel[1], Bhanu Bhakta Neupane[2,3], Basant Giri[2*]

[1]Birendra Multiple Campus, Tribhuvan University, Chitwan, Nepal

[2]Center for Analytical Sciences, Kathmandu Institute of Applied Sciences, Kathmandu, Nepal

[3]Amrit Campus, Tribhuvan University, Kathmandu, Nepal

*Corresponding author

Email: bgiri@kias.org.np

PO BOX 23002

Kathmandu, Nepal

Phone: 0977-01-6924204

S1 File: Sample questionnaire

This survey is a part of our research work that aims to understand the chemistry laboratory safety practices and issues in Nepal.

Currently, we focus only on teaching labs. Fill up this questionnaire only if you are teaching or supervising chemistry lab in +2 or higher level.

Please provide accurate response to the questions at your best level.

If you are involved in more than one institution, please consider the one in which you spend more time.

Information collected in this survey will be used only for research purpose. Results may be available to public via scientific publication and reports. Institutional and/or personal information will not be shared to third party.

If you do not wish to participate in this survey, please just ignore.

We appreciate your cooperation.

Randomly selected three respondents will receive NRs 2500 each.
****

General questions

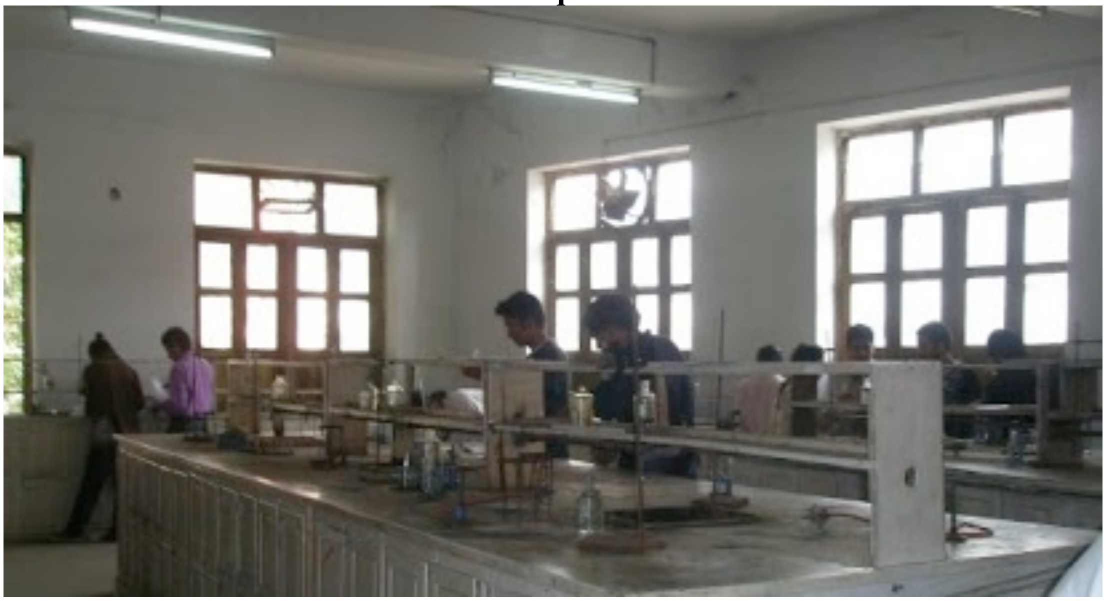

1. Do you teach/supervise chemistry laboratory classes?
   a. No
   b. Yes
2. The chemistry laboratory you are associated with is used for (you may select multiple answers if applicable)
   a. High school (+2)/A level
   b. Bachelors (undergraduate)
   c. Masters (graduate)
   d. Other (specify):
3. How many students are allowed to work in one laboratory session?
   a. < 10
   b. 10-20

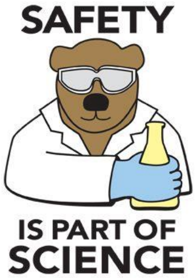

c. 21-30
d. 31-40
e. > 40

4. How many instructors/teachers/teaching assistants/lab assistants in each lab session?
   a. 0
   b. 4
   c. 1
   d. 3
   e. 2
5. Size of the chemistry laboratory in your institution is (in square meter, m^2)
   a. > 80
   b. 41-60
   c. < 20
   d. 21-40
   e. 61-80

Thank you for starting the survey. In the following section, we will ask questions related to equipment and practices used for safety in chemistry lab.

Laboratory safety equipment and practices

1. Students can start their lab without any safety orientation.
   a. Agree
   b. Disagree
2. A written safety policy is explained to students.
   a. Agree
   b. Disagree
3. Students sign a form acknowledging they understood the safety rules and regulations before the start of laboratory session.
   a. Agree
   b. Disagree

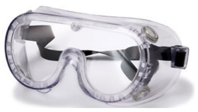
Safety goggles

4. Students wear lab coat (apron) during laboratory session.
   a. Agree
   b. Disagree
5. Students are required to wear safety goggles (shown in photo) for eye protection inside lab.
   a. Agree
   b. Disagree
6. "Proper clothing" as a personal protective equipment (PPE) is enforced in your laboratory. Bare foot, high hill shoes, shoes with open toes, sandals are not safe. Woven materials, shorts, miniskirts are not safe too. Wearing jewelry increases risk.
   a. Agree
   b. Disagree

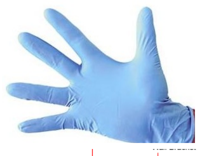

7. Appropriate gloves are used during chemical handling.
   a. Disagree
   b. Agree
8. Food and beverages are not allowed in the lab.
   a. Agree
   b. Disagree

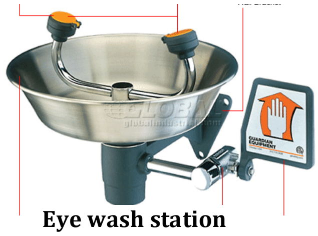
Eye wash station

9. An appropriate eye wash station facility (shown in photo or similar) is NOT available in our laboratory. Eye wash facilities are used to clean eyes when chemicals get into the eye.
   a. Disagree
   b. Agree

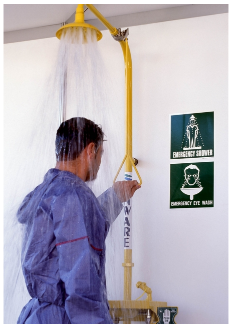

10. Safety shower (shown in photo) is NOT available in the laboratory.
   a. Disagree
   b. Agree

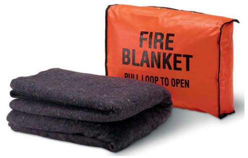

11. There is NO fire extinguisher inside the lab.
   a. Agree   b. Disagree
12. First aid kit is available in the lab.
   a. Agree   b. Disagree

13. There is NO fire blanket (like shown above) present in the lab.
   a. Agree
   b. Disagree
14. Working condition fume hood is available for use in your lab.
   a. Agree
   b. Disagree

Storage of chemicals

In this section, we will ask questions related to the storage of chemicals.

1. A separate stockroom is used to store chemicals.
   a. Agree
   b. Disagree
2. Stockroom is locked and is not generally available to students.
   a. Agree
   b. Disagree
3. The stockroom has a separate ventilating system.
   a. Agree
   b. Disagree
4. The stockroom does not have a water sprinkler system.
   a. Agree
   b. Disagree
5. The stockroom does not have fire/smoke alarm system.
   a. Agree
   b. Disagree
6. A chemical inventory list is maintained and is updated on regular basis.
   a. Agree
   b. Disagree
7. Chemicals are placed in alphabetical order.
   a. Agree
   b. Disagree
8. Acids and bases are kept in separate appropriate acid and base cabinets.
   a. Agree
   b. Disagree

9. Flammable chemicals are stored in appropriate flammables cabinets.
   a. Agree
   b. Disagree
10. Organic solvents are placed in separate cabinet.
   a. Agree
   b. Disagree
11. Gas tanks are chained.
   a. Agree
   b. Disagree
12. MSDS sheets are on the file and readily available to students and teachers. Material Safety Data Sheets (MSDS) contains information regarding the proper procedures for handling, storing, and disposing of chemical substances.
   a. Agree
   b. Disagree
13. Safety manual is available to teachers and students.
   a. Agree b. Disagree

Emergency and accidents

1. Have you ever experienced or seen any hazard/accident while you were working in a chemistry laboratory?
   a. Yes
   b. No
2. If yes, please explain what kind of accident.
3. Your institution has a certified first-aid and CPR person available at any time in case of emergency.
   a. Agree
   b. Disagree
4. You are in contact with local health care center in case of emergency.
   a. Agree
   b. Disagree
   c. I do not know
5. List of hazardous chemicals is available in lab.
   a. Agree
   b. Disagree
6. You have been trained and certified in first-aid and cardiopulmonary resuscitation (CPR).
   a. Agree
   b. Disagree
7. What are the five most hazardous chemicals in your laboratory?
   a.
   b.
   c.
   d.
   e.

Congratulations!
You are 50% done.

Chemical waste management

1. We dump chemical waste directly into the drain/sink.
   a. Depends on chemical
   b. Disagree
   a. Agree
2. Based on the nature of waste chemicals, they are collected in separate containers.
   b. Agree
   c. Disagree
3. Acidic/basic waste is neutralized with appropriate base/acid before dumping into the sink.
   a. Agree
   b. Disagree
4. Acidic and basic wastes are diluted with water before dumping into sink.
   a. Agree
   b. Disagree
5. Organic waste is collected in a separate container.
   a. Agree
   b. Disagree
6. There is a separate container for broken glass and sharp objects.
   a. Agree b. Disagree
7. All laboratory waste is managed at our own institution.
   a. Agree b. Disagree
8. Solid chemical waste is dumped as regular garbage, which is taken away to dumping sites.
   a. Agree b. Disagree

Safety training and policy

1. Students and other lab users undergo an orientation program on safety procedures at the beginning of the class/lab session.
   a. Agree b. Disagree
2. Every new science personnel (e.g., faculty, lab assistant) are required to go through a safety-training program.
   a. Agree b. Disagree
3. Have you ever participated in a training program/course on chemical safety in laboratories?
   a. No b. Yes
4. My educational training provided me with the experience needed to safely handle, store, and dispose off chemicals in teaching setting.
   a. Agree b. Disagree
5. I never learned how to safely handle, store, or dispose of chemicals.
   a. Agree b. Disagree

6. Training on the safe handling, storage, and disposal of chemicals should be taught as a part of the collegiate educational training of chemistry teachers.
   a. Agree b. Disagree
7. My students and I would benefit from a short course or workshop focusing on the safe handling, storage, and disposal of chemicals.
   a. Agree b. Disagree
8. Only one person is responsible for purchasing chemicals in our institution.
   a. Agree b. Disagree
9. If there is online safety course, I would be more apt to enroll for this course.
   a. Agree b. Disagree

Monitoring and evaluation

1. The government does not monitor the safety issues in your lab.
   a. Agree b. Disagree
2. Eye wash station is inspected on a regular basis.
   a. Agree b. Disagree
3. One can buy any chemical without prior approval from government agencies.
   a. Agree b. Disagree
4. Your institution carries out regular safety inspection of lab.
   a. Agree b. Disagree
5. There is no government body to monitor safety issues in our lab.
   a. Agree b. Disagree
6. The safety shower is inspected on a regular basis.
   a. Agree b. Disagree

Concluding questions

1. You have been teaching chemistry for past …….years.

2. The highest degree you have earned is
   a. High school (+2/A level) b. Bachelor c. MSc
   b. MPhil d. PhD

3. You school/college is located in ……………………………district.

Optional questions (for a chance to win NRs 2500)

Your name: Your email:

Contact information

Mr. Krishna Kandel<br>Birendra Multiple Campus, Chitwan, Nepal<br>kandelkrishnaji@gmail.com<br>9855060428	Dr. Basant Giri<br>Center for Analytical Sciences<br>Kathmandu Institute of Applied Sciences<br>Kathmandu, Nepal<br>bgiri@kias.org.np<br>9843677021
