# Supplementary material for: Status of chemistry lab safety in Nepal
Source: PLoS One. 2017 Jun 23;12(6):e0179104. doi: 10.1371/journal.pone.0179104 (PMC5482429; doi:10.1371/journal.pone.0179104)
Supplement: S2 File — (PDF) [file pone.0179104.s002.pdf]

Status of chemistry lab safety in Nepal

Krishna Prasad Kandel[1], Bhanu Bhakta Neupane[2,3], Basant Giri[2*]

[1]Birendra Multiple Campus, Tribhuvan University, Chitwan, Nepal

[2]Center for Analytical Sciences, Kathmandu Institute of Applied Sciences, Kathmandu, Nepal

[3]Amrit Campus, Tribhuvan University, Kathmandu, Nepal

*Corresponding author

Email: bgiri@kias.org.np

PO BOX 23002

Kathmandu, Nepal

Phone: 0977-01-6924204

S2 File: Data details

S2.1 Background information of respondents

a. The highest degree you have earned is:

Degree	Number of responses
High school (+2)	3
Bachelor	4
Master	122
Ph.D.	9

b. You have been teaching chemistry for past …….years

Years	Number of responses
<6	40
6-10	64
11-15	12
16-20	9
>20	5

c. The chemistry laboratory is used for:

Program	Number of responses
High school (+2, A level)	56
Bachelors	37
High school + Bachelor	28
Masters	11
Bachelors/Masters	2
All	2

d. How many students are allowed to work in one laboratory session?

Student number	Number of responses
<10	3
11-20	68
21-30	52
31-40	10
>40	5

e. How many instructors/teachers/teaching assistants/lab assistants in each lab session?

Number of instructors	# of responses
1	17
2	70
2	41
4	10

f. Size of the chemistry laboratory (in square meter) in your institution

Size of lab (m^2)	Number of responses
<20	11
21-40	60
41-60	26
61-80	12
>80	28

S2.2 Response to various questions on lab safety equipment and practices

Question	Response	
	Agree	Disagree
Safety manual is available to teachers and students	64	73
MSDS sheets are on the file and readily available to students and teachers	35	100
Working condition fume hood is available for use in lab	60	76
There is NO fire extinguisher inside the lab	64	70
First aid kit is available in lab	92	46
An appropriate eye wash station facility (shown above or similar) is NOT available	74	64
Gloves are used during chemical handling	78	58
Students are required to wear safety goggles for eye protection inside laboratory	86	51
Food and beverages are not allowed in lab	106	23

S2.3 Response to questions related to chemical storage practices

Question	Response Agree	Disagree
A chemical inventory list is maintained and is updated on regular basis	78	59
Acids and bases are kept in separate appropriate acid and base cabinets	92	44
Flammable chemicals are stored in appropriate flammables cabinets	60	76
A separate stockroom is used to store chemicals	121	17

S2.4 Responses on emergency handling questions

(a) Percentage of respondents that agreed and disagreed on the given statement

Questions	Agree	Disagree
Witnessed/experienced any hazards/accidents in the laboratory	65	73
Certified first-aid and cardiopulmonary resuscitation (CPR) person available	28	110

(b) Number of times the given accident occurred

Type of accident	# of times reported
Acid burning skin and cloth	16

Organic solvent corrosion	8
Sodium metal/water explosion	4
Hydrogen gas related explosion/burning	4
Inhaling gas (NH_3, H_2S, SO_2)	3
Glass cut	5
Fire due to organic solvents/LPG gas	2

(c) The most frequently reported hazardous chemicals per respondents' knowledge

Hazardous chemicals	# of times reported
Sulfuric acid	53
Chloroform	51
Nitric acid	25
Hydrochloric Acid	25
Mercury	21
Formaldehyde	21
Ammonia	17
Hydrogen sulfide	16
Benzene	12
Ether	10
Benzoyl chloride	10
Sodium Hydroxide	7
Lead	6
Toluene	5
Silver nitrate	5
Methanol	5
Thionyl chloride	4
Sodium cyanide	4
Hydrofluoric acid	4
Acetone	4
Sulfur dioxide	3
Sodium metal	3
Phenol	3
Mercury chloride	3
Mercuric chloride	3
Hydrazine	3
Cyanide	3
Arsenic	3
Sodium	2
Hexane	2
Chlorobenzene	2
Carbon tetrachloride	2
Bromine	2
Benzyl Chloride	2

Arsenic oxide	2
Trifluoroacetic acid	1
Pyridine	1
Potassium thiocyanate	1
Potassium permanganate	1
Potassium dichromate	1
Potassium cyanide	1
Nitrobenzene	1
Methyl isocyanate	1
Mercurous nitrate	1
Lead acetate	1
Ethanol	1
Dimethyl formamide	1
Cobalt Nitrate	1
Chromic acid	1
Carbon monoxide	1
Carbon dioxide	1
Calcium oxide	1
Benzaldehyde	1
Aniline	1
Ammonium Hydroxide	1
Aluminum chloride	1
Alumina	1
Acetylene	1
Acetic anhydride	1
Acetic acid	1

S2.5 Responses to waste management questions

Questions	Agree	Disagree	Depends
Solid chemical waste is dumped as regular garbage	99	26	
There is a separate container for broken glass	105	33	
Organic waste is collected in a separate container	58	80	
Acidic/basic waste is neutralized with appropriate base/acid before dumping into the sink	44	92	
Chemical waste is directly dumped into the drain/sink	67	28	42

S2.6 Responses to safety training and policy section

Questions	Agree	Disagree
The government does not monitor the safety issues in lab	87	13
Would benefit from a short course or workshop focusing on the safe handling, storage, and disposal of chemicals	121	17
Training on the safe handling, storage, and disposal of chemicals should be	116	22

taught as a part of the collegiate educational training of chemistry teachers		
Never learned how to safely handle, store, or dispose of chemicals	22	115
Students can start their lab without any safety orientation	56	81
